# Psychotropic medication use and risks of kidney injury and fall-related events: a propensity score–matched cohort study

**DOI:** 10.3389/fphar.2025.1690792

**Published:** 2026-01-12

**Authors:** Yu-Hsien Wu, Shang-Feng Tsai, Shiau-Shian Huang, Po-Hsun Hou

**Affiliations:** 1 Department of Psychiatry, Taichung Veterans General Hospital, Taichung, Taiwan; 2 Division of Clinical Informatics, Department of Digital Medicine, Taichung Veterans General Hospital, Taichung, Taiwan; 3 Division of Nephrology, Department of Internal Medicine, Taichung Veterans General Hospital, Taichung, Taiwan; 4 Department of Post-Baccalaureate Medicine, College of Medicine, National Chung Hsing University, Taichung, Taiwan; 5 Department of Life Science, Tunghai University, Taichung, Taiwan; 6 College of Medicine, National Yang Ming Chiao Tung University, Taipei, Taiwan; 7 School of Public Health, National Defense Medical Center, Taipei, Taiwan; 8 Department of Psychiatry, Taipei Veterans General Hospital, Taipei, Taiwan; 9 Nankung Psychiatric Hospital, Keelung, Taiwan

**Keywords:** acute kidney injury, advanced chronic kidney disease, falls, fracture, psychotropic medications, TriNetX

## Abstract

**Background:**

Psychotropic medications are widely prescribed, yet their long-term renal and injury-related safety in real-world populations remains incompletely defined. We examined the associations between psychotropic medication use and long-term risks of kidney injury and fall-related adverse events.

**Methods:**

We conducted a retrospective propensity score–matched cohort study using the TriNetX global federated health research network. Adults aged ≥18 years with continuous psychotropic medication use were matched 1:1 to nonusers. Primary outcomes were 5-year acute kidney injury (AKI) and chronic kidney disease (CKD). Secondary outcomes included falls, fractures, head injury, dizziness or vertigo, and rhabdomyolysis. Relative risks (RRs) and 95% confidence intervals (CIs) were estimated.

**Results:**

Among 169,309 matched pairs (mean age 47 years; 69% female), psychotropic medication use was associated with higher 5-year risks of AKI (2.85% vs. 1.55%; RR 1.83, 95% CI 1.75–1.92), incident CKD stage 3 (7.50% vs. 6.18%; RR 1.21, 95% CI 1.18–1.25), and advanced CKD (1.13% vs. 0.88%; RR 1.28, 95% CI 1.20–1.37). Increased risks were also observed for fall-related outcomes, including falls (RR 2.32), fractures (RR 1.85), head injury (RR 1.88), dizziness or vertigo (RR 1.81), and rhabdomyolysis (RR 3.71). Associations were generally consistent across medication classes, age groups, and sex.

**Conclusion:**

Psychotropic medication use was associated with modest but significantly higher long-term risks of kidney injury and fall-related events in a large real-world cohort, even after accounting for underlying psychiatric and neurologic conditions. These findings support the need for integrated renal monitoring and injury-prevention strategies in patients receiving long-term psychotropic therapy.

## Highlights

- In this large real-world cohort study, we evaluated the long-term renal and injury-related safety of psychotropic medications, including antidepressants, using a global federated health research network.
- In a propensity score–matched analysis of more than 330,000 adults (169,309 matched pairs), psychotropic medication use was associated with modest but significantly higher 5-year risks of acute kidney injury, CKD progression (including incident CKD stage 3 and advanced CKD), and fall-related outcomes such as falls, fractures, head injury, dizziness, and rhabdomyolysis.
- These findings highlight the importance of integrated pharmacovigilance, routine kidney function monitoring, and injury-prevention strategies for patients receiving long-term psychotropic therapy, particularly those with elevated baseline risk or clinical complexity.

## Introduction

Psychotropic medications, particularly antidepressants, are among the most widely prescribed medications globally, with increasing use not only for major depressive disorder and anxiety but also for off-label indications such as chronic pain, sleep disorders, and somatic symptom management (Kovich et al., 2023; Olfson et al., 2014; Pratt et al., 2017; Cao et al., 2021; Wong et al., 2017). Their utilization is especially prevalent in older adults and patients with chronic comorbidities including hypertension, diabetes, and chronic kidney disease (CKD) (Vicente et al., 2015; Coupland et al., 2011; Darowski et al., 2009; Zhu et al., 2024). While the therapeutic benefits of psychotropic medications are well established, concerns remain regarding their long-term safety, especially in medically complex populations (Coupland et al., 2011; Darowski et al., 2009; Zhu et al., 2024; Khawam et al., 2006; Brannstrom et al., 2019; Sarwar et al., 2025; Dev et al., 2014; Winkelmayer et al., 2011; Iwaga et al., 2017; Snyder and Kish, 2016).

Multiple classes of psychotropic agents—including selective serotonin reuptake inhibitors (SSRIs), serotonin-norepinephrine reuptake inhibitors (SNRIs), and tricyclic antidepressants (TCAs)—have been associated with adverse effects such as hyponatremia, orthostatic hypotension, and an increased risk of falls and fractures (Viramontes et al., 2016; Carvalho et al., 2016; De Picker et al., 2014). These adverse effects are particularly relevant to patients with CKD or those receiving concomitant medications such as diuretics or nonsteroidal anti-inflammatory drugs (NSAIDs), which may exacerbate nephrotoxicity or impair perfusion (Zhu et al., 2024; De Picker et al., 2014; Balogun et al., 2012; Matsuura et al., 2025). Moreover, potential mechanisms for renal injury have been proposed, including serotonin-mediated vascular effects and drug-induced rhabdomyolysis (Snyder and Kish, 2016; Zhu et al., 2022; Cottone and Chiavarino, 2023; Cana Ruiu et al., 2024).

Psychotropic medications have also been consistently linked to fall-related outcomes through mechanisms such as sedation, impaired balance, orthostatic hypotension, and electrolyte disturbances (Viramontes et al., 2016; Carvalho et al., 2016; De Picker et al., 2014). Importantly, several of these pathways—particularly hemodynamic instability and autonomic dysfunction—may also contribute to renal vulnerability, especially in older adults and patients with multimorbidity. However, prior studies have largely examined renal outcomes and fall-related complications in isolation, limiting the ability to comprehensively characterize the long-term safety profile of psychotropic medications in real-world clinical settings (Khawam et al., 2006; Brannstrom et al., 2019; Sarwar et al., 2025; Dev et al., 2014; Winkelmayer et al., 2011; Iwaga et al., 2017).

Despite widespread use of psychotropic medications, evidence regarding their direct association with acute kidney injury (AKI) or CKD progression remains sparse and inconsistent. Most existing studies have evaluated renal and neurologic complications separately, often without long-term follow-up (Khawam et al., 2006; Brannstrom et al., 2019; Sarwar et al., 2025; Dev et al., 2014; Winkelmayer et al., 2011; Iwaga et al., 2017). Fall-related injuries such as fractures and head trauma are critical safety outcomes due to their associated morbidity, hospitalization, and disability, particularly among older adults (Coupland et al., 2011; Darowski et al., 2009; Brannstrom et al., 2019). To address this gap in post-marketing surveillance, we conducted a large-scale, real-world cohort study using the TriNetX global federated health research network. The TriNetX Global Collaborative Network includes health systems from North America, Europe, the Middle East, and Asia-Pacific, providing access to de-identified electronic health records from more than 15 countries, thereby allowing evaluation of long-term safety outcomes across diverse populations and healthcare settings.

The objective of this study was to evaluate the long-term associations between psychotropic medication use and clinically relevant renal and injury-related outcomes, including AKI, CKD progression (stage 3 and advanced CKD), and fall-related complications. The analysis primarily focused on antidepressants (SSRIs, SNRIs, and other agents), while also including commonly prescribed hypnotics and antipsychotics. Our findings aim to inform ongoing pharmacovigilance and support clinical decision-making regarding the safe use of psychotropic medications in high-risk populations.

## Materials and methods

### Study design and data source

We conducted a retrospective cohort study using the TriNetX global federated health research network, which provides access to de-identified electronic health records (EHRs) from over 122 million individuals across 15 countries. TriNetX has supported pharmacovigilance research and post-marketing safety studies across multiple therapeutic areas, with data structures aligned to support observational pharmacoepidemiology investigations. The network includes hospitals, primary care units, and specialty centers, capturing data from insured and uninsured populations. The TriNetX platform enables real-time analytics through federated queries without transferring patient-level data, thereby preserving data privacy and regulatory compliance (Chen et al., 2023). Available data include demographics, diagnoses (ICD-10-CM), procedures (ICD-10-PCS, CPT), medications (Veterans Affairs National Formulary), laboratory tests (LOINC), and clinical terminology (SNOMED CT).

### Ethics statement

TriNetX adheres to the Health Insurance Portability and Accountability Act (HIPAA) and the General Data Protection Regulation (GDPR). All data are de-identified, and informed consent was not required. The Western Institutional Review Board granted a waiver of consent. This study was also approved by the Institutional Review Board of Taichung Veterans General Hospital (Approval No. SE22220A-1, TCVGH).

### Study population

We identified adults aged ≥18 years with at least one clinical encounter between 2005 and 2022 and available diagnosis and laboratory data. Patients were classified according to exposure to psychotropic medications using a new-user design. Individuals with preexisting end-stage kidney disease or a history of kidney transplantation prior to the index date were excluded. To address confounding by indication, baseline psychiatric and neurologic diagnoses were systematically ascertained and incorporated into the analytic framework. The final study population comprised matched cohorts of psychotropic medication users and nonusers.

### Exposure definition

**FIGURE 1:**
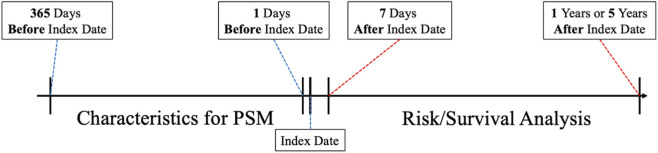
Study timeline for cohort definition and outcome assessment. Schematic of cohort assembly, index date definition, and outcome assessment periods. The index date was assigned based on psychotropic medications prescription criteria (≥3 outpatient or ≥1 inpatient prescriptions and ≥3 months of continuous use). Nonusers had no psychotropic medications exposure. Outcomes were evaluated at 5-year follow-up intervals. PSM indicates propensity score matching.

Psychotropic medication exposure was defined as at least three outpatient visits or one inpatient admission associated with active psychotropic prescriptions and a minimum of 3 months of continuous use, as indicated by uninterrupted medication records. The index date was defined as the first day on which these criteria were fulfilled. Nonusers were defined as individuals with no recorded psychotropic medication prescriptions and at least one clinical encounter during the study period. Both cohorts were followed from their respective index dates. Psychotropic medications were identified using structured medication records based on the Veterans Affairs National Formulary coding system within TriNetX. A schematic timeline of cohort assembly is shown in Figure 1.

### Outcomes

Primary outcomes were the 5-year incidence of AKI and CKD outcomes, including incident CKD stage 3 and advanced CKD. AKI was defined using validated International Classification of Diseases, Tenth Revision, Clinical Modification (ICD-10-CM) diagnostic codes (N17.x), as recorded during inpatient or outpatient clinical encounters. Given the multi-institutional nature of the TriNetX database, uniform serum creatinine–based criteria with standardized time windows could not be consistently applied across participating healthcare organizations. Advanced CKD was defined as CKD stage 4 or 5 (estimated glomerular filtration rate [eGFR] <30 mL/min/1.73 m^2^) or initiation of dialysis. eGFR was calculated using the CKD Epidemiology Collaboration (CKD-EPI) equation. Secondary outcomes included fall-related events, defined as falls, fractures, and head injuries, as well as rhabdomyolysis and dizziness or vertigo, identified using ICD-10-CM diagnostic codes and structured clinical records.

### Covariates

Baseline covariates included age, sex, race/ethnicity, and major comorbidities, including hypertension, diabetes mellitus, hyperlipidemia, atherosclerotic heart disease, chronic obstructive pulmonary disease, and prior stroke. To address confounding by indication, baseline psychiatric and neurologic diagnoses were systematically included, encompassing depressive disorders, anxiety disorders, bipolar disorder, schizophrenia, dementia, and cognitive impairment. Concomitant medications with potential renal effects, including renin–angiotensin system (RAS) inhibitors and sodium–glucose cotransporter 2 (SGLT2) inhibitors, were also included. Laboratory variables included systolic blood pressure, hemoglobin A1c, and serum creatinine.

### Statistical analyses

Propensity score matching was performed using logistic regression to estimate the probability of psychotropic medication exposure, incorporating all baseline covariates. Patients were matched 1:1 using nearest-neighbor matching, and standardized mean differences <0.1 were considered indicative of adequate covariate balance. All outcome analyses were conducted using the matched cohorts derived from the primary propensity score model. Incidence rates, absolute risk differences, relative risks (RRs), and 95% confidence intervals (CIs) were calculated. Relative risks were estimated using Poisson regression models with a log link and robust (sandwich) standard errors. Correlation within matched pairs was accounted for using clustered variance estimators within the TriNetX analytic framework. Time-to-event analyses were evaluated using Kaplan-Meier methods to account for censoring and variable follow-up time. Continuous variables were compared using Student’s t tests, and categorical variables were compared using chi-square tests, as appropriate.

All analyses were performed using the validated, SAS-based analytics environment embedded within the TriNetX platform, which does not permit external coding or export of patient-level data, ensuring standardized and reproducible analytical procedures.

## Results

### Study population and baseline characteristics

**FIGURE 2:**
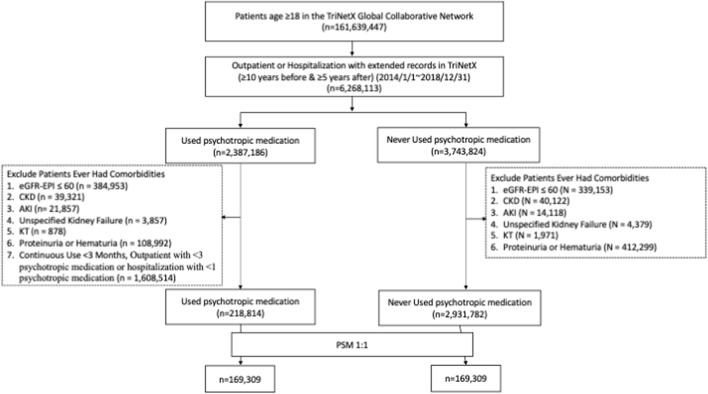
Cohort selection flowchart. Flowchart illustrating cohort selection from the TriNetX database. Adults aged ≥18 years with and without psychotropic medication exposure were identified. Patients with preexisting end-stage kidney disease or a history of kidney transplantation before the index date were excluded. After 1:1 propensity score matching incorporating demographic characteristics, medical comorbidities, and psychiatric and neurologic diagnoses, 169,309 matched pairs were included in the final analytic cohort. Abbreviations: AKI, acute kidney injury; CKD, chronic kidney disease; KT, kidney transplantation.

A total of 218,814 adults with psychotropic medication use and 2,931,782 eligible nonusers were initially identified within the TriNetX network. After applying the inclusion and exclusion criteria and performing 1:1 propensity score matching that incorporated demographic variables, medical comorbidities, and psychiatric and neurologic diagnoses, 169,309 matched pairs of psychotropic medication users and nonusers were included in the final analytic cohort (Figure 2). Baseline characteristics were well balanced between the two groups after matching, with all standardized mean differences <0.1 (Table 1).

**TABLE 1:** Baseline characteristics of psychotropic medication users and nonusers before and after propensity score matching including psychiatric diagnoses.

​	Before matching				After matching			
	Users	Non-users	*p*	SMD	Users	Non-users	*p*	SMD
Total number	218814	2931782	​	​	169309	169309	​	​
Age at index	46.6 ± 17.7	44.6 ± 20.2	<0.001	0.104	47.4 ± 17.6	47.6 ± 18.2	<0.001	0.010
Female, n (%)	153020 (69.93)	1710349 (58.34)	<0.001	0.244	116821 (69.00)	117335 (69.30)	0.056	0.007
Comorbidity								
Hypertensive diseases	45704 (20.89)	132465 (4.52)	<0.001	0.507	29557 (17.46)	31599 (18.66)	0.000	0.031
Hyperlipidemia	25599 (11.70)	83261 (2.84)	<0.001	0.346	16810 (9.93)	17659 (10.43)	0.000	0.017
Diabetes mellitus	16337 (7.47)	51166 (1.75)	<0.001	0.276	10598 (6.26)	11344 (6.70)	0.000	0.018
ASHD	6157 (2.81)	15627 (0.53)	<0.001	0.179	3914 (2.31)	4401 (2.60)	0.000	0.019
COPD	6730 (3.08)	7872 (0.27)	<0.001	0.220	3471 (2.05)	4180 (2.47)	0.000	0.028
Cerebral infarction	2361 (1.08)	3225 (0.11)	<0.001	0.126	1338 (0.79)	1629 (0.96)	0.000	0.018
Other anxiety disorders	44989 (20.56)	11132 (0.38)	<0.001	0.698	12689 (7.50)	11123 (6.57)	0.000	0.036
Depressive episode	35604 (16.27)	7879 (0.27)	<0.001	0.607	8992 (5.31)	7749 (4.58)	0.000	0.034
Bipolar disorder	6260 (2.86)	1412 (0.05)	<0.001	0.237	1608 (0.95)	1403 (0.83)	0.000	0.013
Major depressive disorder, recurrent	9268 (4.24)	1058 (0.04)	<0.001	0.294	1524 (0.90)	1052 (0.62)	0.000	0.032
Schizophrenia	2162 (0.99)	1383 (0.05)	<0.001	0.131	840 (0.50)	708 (0.42)	0.001	0.012
Phobic anxiety disorders	1954 (0.89)	421 (0.01)	<0.001	0.131	478 (0.28)	397 (0.23)	0.006	0.009
Unspecified dementia	1029 (0.47)	438 (0.02)	<0.001	0.093	347 (0.21)	355 (0.21)	0.762	0.001
Mild cognitive impairment	611 (0.28)	281 (0.01)	<0.001	0.071	219 (0.13)	221 (0.13)	0.924	<0.001
Dementia in other diseases classified elsewhere	561 (0.26)	209 (0.01)	<0.001	0.069	162 (0.10)	164 (0.10)	0.912	0.000
Vascular dementia	206 (0.09)	77 (0.00)	<0.001	0.042	67 (0.04)	62 (0.04)	0.660	0.002
Laboratory data								
SBP	123.7 ± 17.3	122.1 ± 17.9	<0.001	0.087	124.1 ± 17.4	123.7 ± 17.7	<0.001	0.024
eGFR-EPI	99.0 ± 18.8	95.7 ± 18.0	<0.001	0.179	98.5 ± 18.7	97.7 ± 18.9	<0.001	0.042
HbA1c	6.1 ± 1.9	6.5 ± 1.5	<0.001	0.200	6.1 ± 1.9	6.4 ± 1.5	<0.001	0.137
Medications, n (%)								
RAS-acting agents	23480 (10.73)	76747 (2.62)	<0.001	0.329	17046 (10.07)	18816 (11.11)	<0.001	0.034
NSAIDs	23119 (10.57)	55907 (1.91)	<0.001	0.364	14538 (8.59)	15649 (9.24)	<0.001	0.023
SGLT2 inhibitors	574 (0.26)	451 (0.02)	<0.001	0.066	251 (0.15)	314 (0.19)	0.008	0.009

Abbreviations: SMD, standardized mean difference; ASHD, atherosclerotic heart disease; COPD, chronic obstructive pulmonary disease; SBP, systolic blood pressure; eGFR, estimated glomerular filtration rate (CKD-EPI, formula). RAS, renin–angiotensin system; NSAIDs, nonsteroidal anti-inflammatory drugs; SGLT2, sodium–glucose cotransporter 2.

The mean age at index date was 47.4 years (SD 17.6) among psychotropic medication users and 47.6 years (SD 18.2) among nonusers. Women accounted for 69.0% of psychotropic medication users and 69.3% of nonusers. The prevalence of major cardiometabolic and vascular comorbidities was comparable between groups, including hypertension (17.5% vs. 18.7%), hyperlipidemia (9.9% vs. 10.4%), diabetes mellitus (6.3% vs. 6.7%), atherosclerotic heart disease (2.3% vs. 2.6%), chronic obstructive pulmonary disease (2.1% vs. 2.5%), and prior cerebral infarction (0.8% vs. 1.0%).

Baseline psychiatric and neurologic conditions, including depressive disorders, anxiety disorders, bipolar disorder, schizophrenia, and dementia-related diagnoses, were also well balanced following matching (Table 1). Concomitant medications with potential renal or hemodynamic effects showed similar distributions between groups, including renin–angiotensin system inhibitors (10.1% vs. 11.1%), nonsteroidal anti-inflammatory drugs (8.6% vs. 9.2%), and sodium–glucose cotransporter 2 inhibitors (0.15% vs. 0.19%). Baseline laboratory parameters were closely aligned, with mean systolic blood pressure of 124.1 vs. 123.7 mmHg, mean estimated glomerular filtration rate of 98.5 vs. 97.7 mL/min/1.73 m^2^, and mean hemoglobin A1c of 6.1% vs. 6.4% among users and nonusers, respectively.

### Primary outcomes: AKI and advanced CKD

**FIGURE 3:**
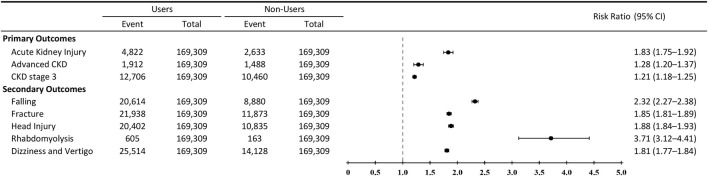
F Forest plot of 5-year relative risks for renal and injury-related outcomes among psychotropic medication users versus matched nonusers. Forest plot displaying 5-year relative risks (RRs) and 95% confidence intervals for adverse clinical outcomes comparing psychotropic medication users with matched nonusers in a propensity score–matched cohort incorporating psychiatric and neurologic diagnoses. Primary outcomes included acute kidney injury, incident CKD stage 3, and advanced CKD (stage 4–5 or dialysis). Secondary outcomes included falls, fractures, head injury, dizziness or vertigo, and rhabdomyolysis. Relative risks were estimated using Poisson regression models. The vertical dashed line indicates the null value (RR = 1.0).

As shown in Figure 3, psychotropic medication use was associated with higher 5-year risks of AKI and CKD outcomes compared with matched nonusers in the propensity score–matched cohort incorporating psychiatric and neurologic diagnoses.

At 5 years, AKI occurred in 4,822 of 169,309 psychotropic medication users (2.85%) and 2,633 of 169,309 nonusers (1.55%), corresponding to a RR of 1.83 (95% CI, 1.75–1.92). With respect to CKD outcomes, psychotropic medication use was associated with increased risks of both incident CKD stage 3 and advanced CKD (stage 4–5 or dialysis). The 5-year incidence of CKD stage 3 was 7.50% among users and 6.18% among nonusers (RR, 1.21; 95% CI, 1.18–1.25), while advanced CKD occurred in 1.13% of users compared with 0.88% of nonusers (RR, 1.28; 95% CI, 1.20–1.37).

Stage-specific analyses indicated that the observed association with CKD outcomes was primarily driven by progression to CKD stage 3 and stage 4, whereas progression to CKD stage 5 or dialysis-requiring kidney failure remained uncommon in both groups.

### Secondary outcomes: falls, fractures, and neurologic complications

As shown in Figure 3, psychotropic medication use was associated with higher 5-year risks of fall-related and injury-related outcomes compared with matched nonusers in the propensity score–matched cohort. At 5 years, falls occurred in 20,614 of 169,309 users (12.18%) and 8,880 of 169,309 nonusers (5.24%), corresponding to an RR of 2.32 (95% CI, 2.27–2.38). Fractures were also more common among users (12.96% vs. 7.01%; RR, 1.85; 95% CI, 1.81–1.89), as were head injuries (12.05% vs. 6.40%; RR, 1.88; 95% CI, 1.84–1.93).

In addition, psychotropic medication users experienced higher rates of neurologic symptoms associated with fall risk. Dizziness or vertigo was more frequent among users (15.07% vs. 8.34%; RR, 1.81; 95% CI, 1.77–1.84). Rhabdomyolysis, although infrequent, occurred more often in users (0.36% vs. 0.10%; RR, 3.71; 95% CI, 3.12–4.41).

### Subgroup analyses by psychotropic medications class, age, and sex

#### Sex-stratified analyses

**FIGURE 4:**
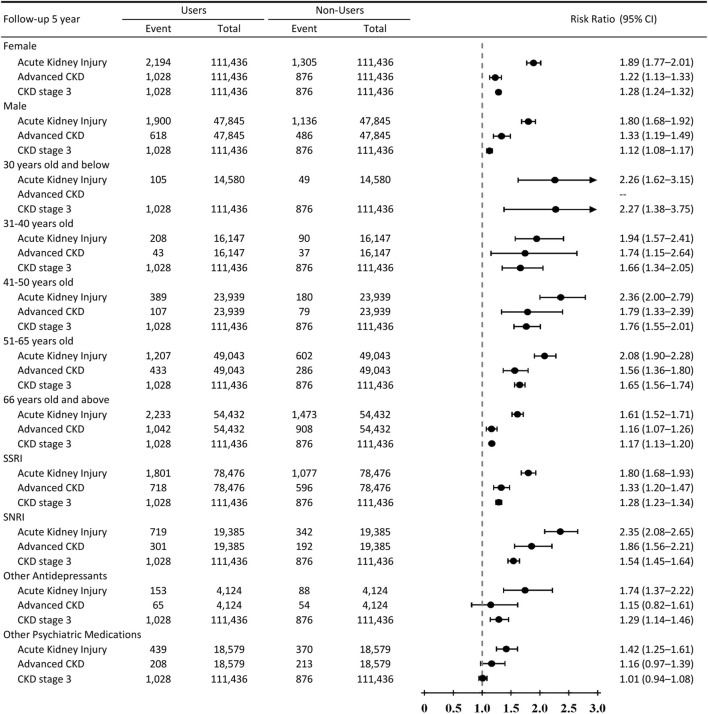
Subgroup analyses of 5-year relative risks for acute kidney injury and CKD outcomes by psychotropic medication class, age, and sex. Forest plot showing 5-year relative risks (RRs) and 95% confidence intervals for acute kidney injury, incident CKD stage 3, and advanced CKD (stage 4–5 or dialysis) among psychotropic medication users compared with matched nonusers, stratified by psychotropic medication class, age group, and sex. Analyses were performed in a propensity score–matched cohort incorporating psychiatric and neurologic diagnoses. Points represent point estimates, horizontal bars denote 95% confidence intervals, and the vertical dashed line indicates the null value (RR = 1.0).

Among women, psychotropic medication use was associated with higher risks of AKI (RR, 1.89; 95% CI, 1.77–2.01), advanced CKD (RR, 1.22; 95% CI, 1.13–1.33), and CKD stage 3 (RR, 1.28; 95% CI, 1.24–1.32). Similar associations were observed among men, including increased risks of AKI (RR, 1.80; 95% CI, 1.68–1.92) and advanced CKD (RR, 1.33; 95% CI, 1.19–1.49), while the association with CKD stage 3 was more modest (RR, 1.12; 95% CI, 1.08–1.17) (Figure 4).

#### Age-stratified analyses

Across age groups, psychotropic medication use was consistently associated with elevated AKI risk, with relative risks ranging from 1.61 (95% CI, 1.52–1.71) among individuals aged ≥66 years to 2.36 (95% CI, 2.00–2.79) among those aged 41–50 years. For advanced CKD, relative risks were generally higher in middle-aged groups (e.g., RR 1.79 [95% CI, 1.33–2.39] in those aged 41–50 years and RR 1.56 [95% CI, 1.36–1.80] in those aged 51–65 years), whereas associations in the youngest age group were limited by low event counts. Associations with CKD stage 3 were observed across age strata, with relative risks ranging from 1.17 to 1.76 (Figure 4).

#### Psychotropic medication class

As shown in Figure 4, when stratified by psychotropic medication class, increased risks of AKI were observed among users of SSRIs (RR, 1.80; 95% CI, 1.68–1.93), SNRIs (RR, 2.35; 95% CI, 2.08–2.65), other antidepressants (RR, 1.74; 95% CI, 1.37–2.22), and other psychiatric medications (RR, 1.42; 95% CI, 1.25–1.61). For advanced CKD, elevated risks were noted particularly among SNRI users (RR, 1.86; 95% CI, 1.56–2.21) and SSRI users (RR, 1.33; 95% CI, 1.20–1.47), whereas associations for other psychiatric medications were more modest and, for CKD stage 3, not statistically significant (RR, 1.01; 95% CI, 0.94–1.08).

Additional time-to-event and class-based analyses were performed to further evaluate the robustness of the findings. Time-to-event Cox proportional hazards models demonstrated consistent 5-year hazard associations between psychotropic medication use and renal and fall-related outcomes (Supplementary Figure S1). In class-based comparisons, SNRI use was associated with higher 5-year hazards of the primary endpoint compared with SSRI use (Supplementary Figure S2).

## Discussion

In this large, real-world, propensity score–matched cohort study, psychotropic medication use was associated with higher 5-year risks of AKI, incident CKD stage 3, advanced CKD, and fall-related adverse outcomes, including fractures, head injuries, dizziness, and rhabdomyolysis (Coupland et al., 2011; Darowski et al., 2009; Brannstrom et al., 2019; Sarwar et al., 2025; Dev et al., 2014). These associations were observed after accounting for a broad range of demographic characteristics, medical comorbidities, and importantly, baseline psychiatric and neurologic diagnoses, suggesting that psychotropic medication use may contribute to long-term renal and injury-related risks in routine clinical care.

By focusing on 5-year outcomes, our analysis emphasizes clinically meaningful long-term risk patterns rather than short-term fluctuations, which is particularly relevant for medications often prescribed chronically. These findings extend prior literature linking psychotropic medications to fall risk (Coupland et al., 2011; Darowski et al., 2009; Brannstrom et al., 2019), and quantify these effects over a multi-year horizon. Previous studies have implicated SSRIs, SNRIs, and tricyclic antidepressants in fall-related events through mechanisms such as sedation, impaired balance, orthostatic hypotension, and electrolyte disturbances (Viramontes et al., 2016; Carvalho et al., 2016; De Picker et al., 2014). Consistent with these reports, our study demonstrates substantially elevated long-term risks of falls, fractures, and head injuries, underscoring the cumulative burden of injury-related complications among psychotropic medication users.

Regarding renal outcomes, our findings align with and extend emerging evidence that psychiatric conditions themselves are associated with increased risks of AKI and CKD progression. A population-based cohort study from Sweden reported that individuals diagnosed with stress-related disorders had elevated risks of both AKI and CKD progression, independent of baseline kidney function (Su et al., 2021). More recently, a large prospective analysis from the UK Biobank demonstrated that a range of psychiatric disorders—including depression, anxiety, stress-related disorders, and psychotic disorders—were associated with a delayed but sustained increase in CKD risk (Li et al., 2025). Together, these studies support the concept that psychiatric morbidity represents an important upstream risk factor for renal vulnerability.

Importantly, in our study, the magnitude of association between psychotropic medication use and renal outcomes was attenuated after incorporating psychiatric and neurologic diagnoses into the primary propensity score model, indicating that part of the observed risk is likely attributable to underlying psychiatric disease burden rather than medication exposure alone. Nevertheless, residual associations persisted, suggesting that medication-related factors—such as hemodynamic effects, autonomic dysregulation, or drug-specific nephrotoxic mechanisms—may further contribute to renal risk.

Proposed mechanisms linking psychotropic medication use to renal injury include serotonin- and norepinephrine-mediated vascular effects, autonomic instability, and susceptibility to AKI episodes that may accelerate CKD progression (Calvi et al., 2021; Ziegler et al., 2025). Recurrent subclinical AKI and injury-related events may represent an important pathway through which long-term renal deterioration occurs, consistent with the observed associations for both AKI and CKD stage progression.

From a clinical perspective, these findings highlight the importance of long-term renal risk awareness when prescribing psychotropic medications, particularly in patients with existing CKD risk factors or those at increased risk of falls. Routine monitoring of kidney function and proactive fall-prevention strategies may help mitigate cumulative renal and injury-related burden in this population. Given the widespread and often prolonged use of psychotropic medications, integrating renal risk considerations into psychiatric care pathways is warranted.

Subgroup analyses indicated that the magnitude of renal risk varied across psychotropic medication classes, with relatively higher risk estimates observed among users of serotonin–norepinephrine reuptake inhibitors and certain non-SSRI antidepressants, as well as among patients receiving other psychiatric medications. However, the overall direction of association was consistent across classes, and age- and sex-stratified analyses demonstrated broadly similar patterns, with larger absolute risk differences observed among middle-aged and older adults.

While previous reports have implicated antipsychotics and hypnotics in renal injury (Calvi et al., 2021; Ziegler et al., 2025), antidepressants such as selective serotonin reuptake inhibitors and serotonin–norepinephrine reuptake inhibitors have traditionally been regarded as relatively renal-safe in clinical practice (Ong et al., 2024). In this context, the subgroup findings in our study should be interpreted cautiously. The observed differences are likely to reflect variation in underlying psychiatric disease severity, treatment indication, and clinical complexity, rather than clear evidence of class-specific nephrotoxicity.

The observed heterogeneity across psychiatric medication classes, particularly the higher hazard estimates associated with SNRIs compared with SSRIs, may reflect differences in pharmacodynamic profiles, including noradrenergic activity and potential effects on hemodynamics and balance. These findings should be interpreted as associative rather than causal.

To further address confounding by indication, psychiatric and neurologic diagnoses were incorporated into the primary propensity score model. After matching, covariates—including psychiatric and neurologic conditions—were well balanced, and the 5-year risks of AKI, CKD outcomes, and fall-related events remained directionally consistent. These findings suggest that the associations observed cannot be fully attributed to underlying psychiatric disease burden alone, while acknowledging that residual confounding may persist.

Nevertheless, unmeasured aspects of psychiatric illness severity, functional impairment, or treatment complexity could not be completely accounted for in this observational study. Accordingly, confounding by indication remains a potential limitation, particularly for injury-related outcomes, and the findings should be interpreted as associative rather than causal.

This study leveraged a large, multinational real-world dataset and applied rigorous propensity score matching to enhance internal validity and generalizability. The inclusion of both biochemical renal outcomes and clinically relevant injury-related endpoints provides a comprehensive assessment of psychotropic medication–associated risks. Moreover, the combined use of time-to-event analyses and relative risk estimation across multiple outcomes strengthens the robustness and interpretability of the findings.

Several limitations merit consideration. As an observational study, residual confounding, including confounding by indication, cannot be fully excluded, despite comprehensive propensity score matching and the inclusion of psychiatric and neurologic diagnoses (Balogun et al., 2012). Granular data on medication dose, duration, adherence, and cumulative exposure were unavailable, precluding dose–response or duration–response analyses. In addition, laboratory data completeness varied across institutions, and early-stage CKD may have been underdetected. Accordingly, causal inference is limited, and the associations observed should be interpreted as hypothesis-generating rather than causal.

## Conclusion

Psychotropic medication use was associated with higher long-term risks of AKI, CKD progression, and fall-related complications in a large real-world cohort, even after accounting for underlying psychiatric disease burden. These findings, together with accumulating evidence linking psychiatric disorders to kidney disease, underscore the need for integrated psychiatric–renal risk assessment and long-term monitoring strategies in clinical practice.

## Data Availability

The datasets presented in this article are not readily available because the data that support the findings of this study are available from TriNetX, a global federated research network. Restrictions apply to the availability of these data, which were used under institutional license for this study. Data are not publicly available, but researchers with access to TriNetX may reproduce the analysis. Requests to access the datasets should be directed to ux12091666@gmail.com.
