# Supplementary material for: Psychotropic medication use and risks of kidney injury and fall-related events: a propensity score–matched cohort study
Source: Front Pharmacol. 2026 Jan 12;16:1690792. doi: 10.3389/fphar.2025.1690792 (PMC12833078; doi:10.3389/fphar.2025.1690792)

**Supplementary figures**

**Figure S1. Forest Plot of 5-Year Hazard Ratios for Adverse Clinical Outcomes Associated with Psychotropic Medication Use**

Forest plot showing 5-year hazard ratios (HRs) and 95% confidence intervals (CIs) for renal and fall-related outcomes associated with psychotropic medication use. Hazard ratios were estimated using Cox proportional hazards models to account for time-to-event outcomes. Models were adjusted for demographic characteristics, baseline comorbidities, laboratory parameters, and psychiatric and neurologic diagnoses, consistent with the primary analysis. This analysis was performed to evaluate the robustness of the main findings in a time-to-event framework.


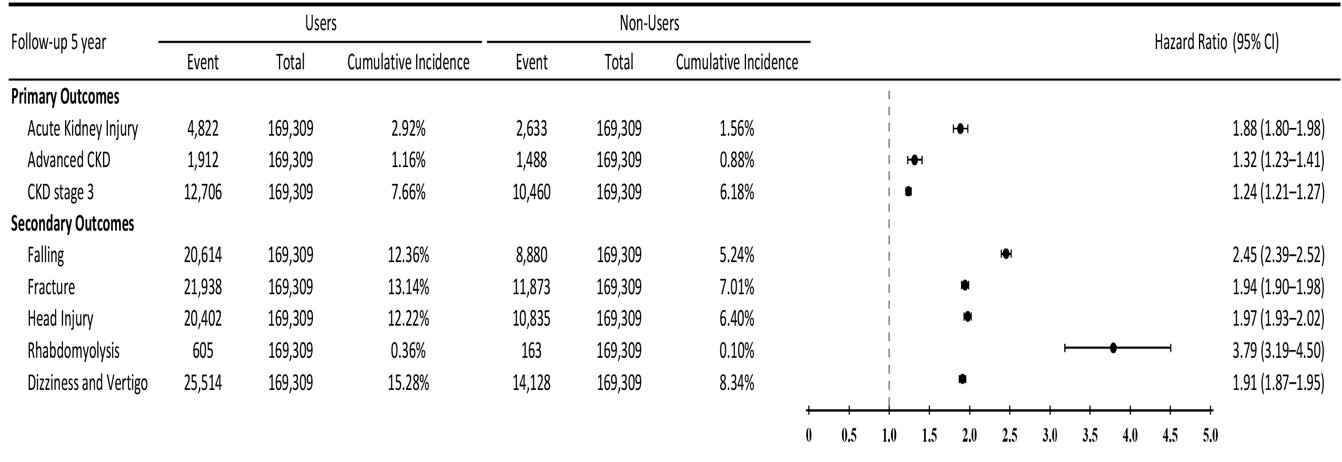


**Figure S2. Forest Plot of 5-Year Hazard Ratios for Adverse Clinical Outcomes Comparing SNRI and SSRI Use**

Forest plot showing 5-year hazard ratios (HRs) and 95% confidence intervals (CIs) for renal and fall-related outcomes comparing serotonin–norepinephrine reuptake inhibitor (SNRI) use with selective serotonin reuptake inhibitor (SSRI) use. Hazard ratios were estimated using Cox proportional hazards models adjusted for the same covariates as in the primary analysis. This class-based comparison was conducted to explore heterogeneity in outcome risks across psychiatric medication classes.


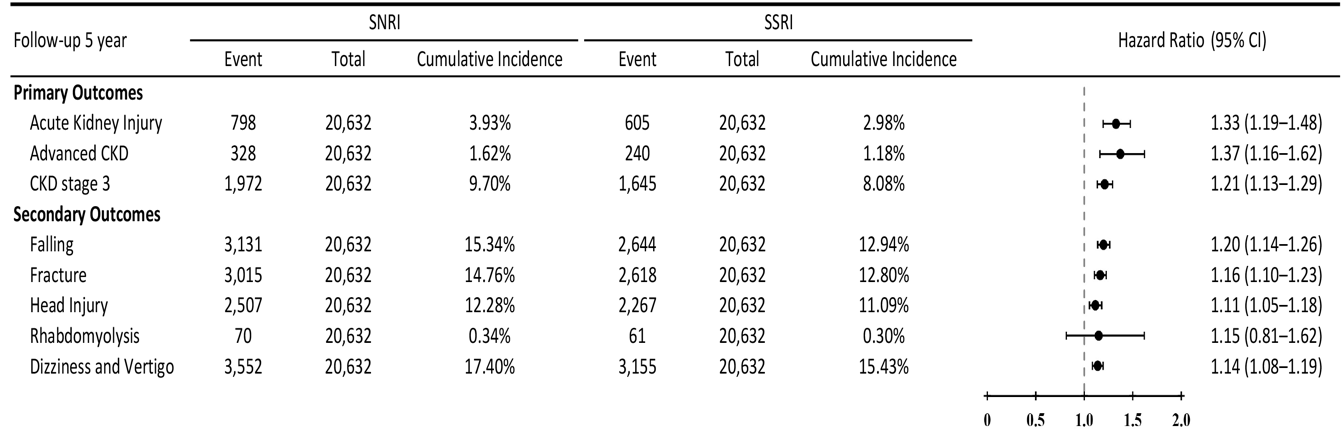

Supplement: Supplementary file 1 [file Supplementaryfile1.docx]
